# [^68^Ga]Ga-DATA^5m^-LM4, a PET Radiotracer in the Diagnosis of SST_2_R-Positive Tumors: Preclinical and First Clinical Results

**DOI:** 10.3390/ijms232314590

**Published:** 2022-11-23

**Authors:** Panagiotis Kanellopoulos, Berthold A. Nock, Lukas Greifenstein, Richard P. Baum, Frank Roesch, Theodosia Maina

**Affiliations:** 1 Molecular Radiopharmacy, INRaSTES, NCSR “Demokritos”, GR-15310 Athens, Greece; 2 CURANOSTICUM Wiesbaden-Frankfurt, DKD Helios Klinik, D-65191 Wiesbaden, Germany; 3 Department Chemie, Standort TRIGA, Johannes Gutenberg-Universität Mainz, D-55126 Mainz, Germany

**Keywords:** somatostatin receptor antagonist, hybrid chelator, Ga-68 labeling, Ga-67 labeling, PET-radiotracer, PET diagnostic imaging

## Abstract

Radiolabeled somatostatin subtype 2 receptor (SST2R)-antagonists have shown advantageous profiles for cancer theranostics compared with agonists. On the other hand, the newly introduced hybrid chelator (6-pentanoic acid)-6-(amino)methyl-1,4-diazepinetriacetate (DATA^5m^) rapidly binds Ga-68 (t_1/2_: 67.7 min) at much lower temperature, thus allowing for quick access to “ready-for-injection” [^68^Ga]Ga-tracers in hospitals. We herein introduce [^68^Ga]Ga-DATA^5m^-LM4 for PET/CT imaging of SST_2_R-positive human tumors. LM4 was obtained by 4Pal^3^/Tyr^3^-substitution in the known SST_2_R antagonist LM3 (H-DPhe-c[DCys-Tyr-DAph(Cbm)-Lys-Thr-Cys]-DTyr-NH_2_) and DATA^5m^ was coupled at the N-terminus for labeling with radiogallium (Ga-67/68). [^67^Ga]Ga-DATA^5m^-LM4 was evaluated in HEK293-SST_2_R cells and mice models in a head-to-head comparison with [^67^Ga]Ga-DOTA-LM3. Clinical grade [^68^Ga]Ga-DATA^5m^-LM4 was prepared and injected in a neuroendocrine tumor (NET) patient for PET/CT imaging. DATA^5m^-LM4 displayed high SST_2_R binding affinity. [^67^Ga]Ga-DATA^5m^-LM4 showed markedly higher uptake in HEK293-SST_2_R cells versus [^67^Ga]Ga-DOTA-LM3 and was stable in vivo. In HEK293-SST_2_R xenograft-bearing mice, it achieved longer tumor retention and less kidney uptake than [^67^Ga]Ga-DOTA-LM3. [^68^Ga]Ga-DATA^5m^-LM4 accurately visualized tumor lesions with high contrast on PET/CT. In short, [^68^Ga]Ga-DATA^5m^-LM4 has shown excellent prospects for the PET/CT diagnosis of SST_2_R-positive tumors, further highlighting the benefits of Ga-68 labeling in a hospital environment via the DATA^5m^-chelator route.

## 1. Introduction

The advent of radiolabeled somatostatin analogs targeting the somatostatin subtype 2 receptor (SST_2_R), one frequently and highly expressed in neuroendocrine tumors (NETs), has revolutionized the management of NET patients [1,2,3]. Accordingly, diagnosis and staging of NETs with positron emission (PET) or single photon emission computed tomography (SPECT) using somatostatin analogs has enabled a selection of patients eligible for subsequent therapy with particle emitters to eradicate SST_2_R-positive tumor lesions. Furthermore, PET or SPECT imaging has been instrumental in addressing important therapy-related questions on dosimetry, therapy planning and post-treatment assessment of therapeutic efficacy. Clearly, such an intertwined diagnosis and therapy, “theranostic”, approach excellently harmonizes with modern precision medicine principles [4,5]. Until recently, octapeptide analogs of native somatostatin-14 (SS-14), such as Tyr^3^-octreotate (TATE, H-DPhe-c[Cys-Tyr-DTrp-Lys-Thr-Cys]-Thr-OH) and Tyr^3^-octreotide (TOC, H-DPhe-c[Cys-Tyr-DTrp-Lys-Thr-Cys]-Thr(ol)), have been properly modified to accommodate radiometals of clinical interest for diagnosis (SPECT—Tc-99m, In-111; PET: Ga-68, Cu-64) [6,7,8,9] and therapy (Lu-177, Ac-225, Pb-212) [10,11,12]. The theranostic pair [^68^Ga]Ga/[^177^Lu]Lu-DOTA-TATE (DOTA, 1,4,7,10-tetraazacyclododecane-N,N′,N″,N‴-tetraacetic acid) represents a successful paradigm of this approach, having been approved by the United States Food and Drug Agency (FDA) and the European Medicines Agency (EMA) for treatment of human SST_2_R-positive tumors [3,13].

**Figure 1:**
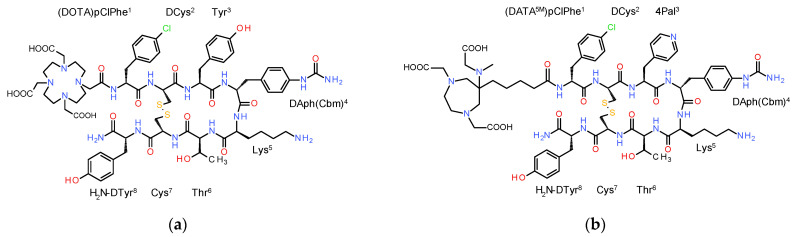
Chemical structures of (**a**) DOTA-LM3; (**b**) DATA^5m^-LM4; the two analogs differ in the chelator and in the residue at position 3: Tyr (in LM3) and (4-pyridyl)alanine (4Pal, in LM4).

The above class of octreotide-derived radioligands has shown agonistic properties at the SST_2_R, associated with fast internalization in target cells after binding to the cell membrane receptor. In fact, high radioligand uptake and retention in tumor lesions have been directly linked to fast internalization in cancer cells [7,8]. In the course of these developments, though radiolabeled octapeptides with antagonistic properties at the SST_2_R, it turned out to perform better than agonists [14,15,16,17,18,19]. Despite their lack of internalization, radiolabeled SST_2_R-antagonists displayed higher and more persistent uptake in tumor-bearing mice combined with faster background clearance. Such properties were next translated in NET patients, challenging the initial reading of SST_2_R-agonism as a major element of the success for somatostatin radioligands. Studies at the molecular and cellular level revealed that radiolabeled SST_2_R-antagonists bind to both active and inactive conformations of the receptor. Hence, antagonists have a far higher number of binding sites at their disposal than agonists, binding to the sub-population of active SST_2_Rs only [20]. A fair number of SST_2_R-antagonists have been developed in recent years carrying suitable chelators for labeling with radiometals of interest [21,22]. Such an example is DOTA-LM3 (DOTA-DPhe-c[DCys-Tyr-DAph(Cbm)-Lys-Thr-Cys]-DTyr-NH_2_; DAph(Cbm), D-4-(carbamoyl)amino-phenylalanine) (Figure 1). DOTA-LM3 labeled with Ga-68 and other radiometals has shown promising qualities during preclinical and clinical studies for use in NET theranostics [21,22].

It should be noted however that incorporation of Ga-68 to DOTA requires heating, complicating hospital logistics in view of the short half-life of Ga-68 (t_1/2_: 67.7 min) [23,24]. An elegant way to tackle this problem is provided by the hybrid chelator (6-pentanoic acid)-6-(amino)methy-1,4-diazepinetriacetate (DATA^5m^). DATA^5m^ rapidly coordinates Ga-68 at much lower temperature, thus allowing for quick and convenient access to “ready-for-injection” [^68^Ga]Ga-tracers in hospitals [25,26,27,28,29,30]. We herein present [^68^Ga]Ga-DATA^5m^-LM4, as a new candidate for PET/CT imaging of SST_2_R-positive tumors. LM4 is a 4Pal^3^/Tyr^3^-analog of LM3 [21,22,31,32] (4Pal^3^, 3-(4-pyridyl)alanine), which was coupled to DATA^5m^ (Figure 1). In the present work, DOTA-LM3 and DATA^5m^-LM4 were labeled with the longer-lived Ga-67 (t_1/2_: 78.3 h) surrogate for convenient and direct comparison of their preclinical profiles in HEK293-SST_2_R cells and HEK293-SST_2_R tumor-bearing mice. Moreover, clinical grade [^68^Ga]Ga-DATA^5m^-LM4 was easily produced in a hospital setting and evaluated in a NET patient applying PET/CT in a “proof-of-principle” approach.

## 2. Results

### 2.1. Ligands and Radioligands

Labeling of the new DATA^5m^-LM4 analog with Ga-67 was accomplished within 15 min at room temperature affording the [^67^Ga]Ga-DATA^5m^-LM4 in high radiochemical purity (RCP > 96%) at a molar activity of 3.7 MBq/nmol, as verified by radioanalytical HPLC methods (Supplementary Materials Figure S1). Similar results could be obtained with the DOTA-derivatized analogs DOTA-TOC and DOTA-LM3 only by heating of the labeling reaction mixture for 30 min at 90 °C. Radioligands were used as such in all further experiments without purification; the respective solutions were tested before and after all in vitro assays and animal studies (analytical data for DATA^5m^-LM4 and DOTA-LM3 in Table S1, Supplementary Materials).

Labeling of DATA^5m^-LM4 with Ga-68 for clinical use was conducted by an automatic system. Reaction at 50 °C provided RCP ≥ 98%. The automated synthesis led to activities between 413 MBq and 596 MBq for 50 µg of DATA^5m^-LM4 (corresponding to 12.5–18 MBq/nmol molar activity; Supplementary Materials Figure S3). 

### 2.2. In Vitro Studies

#### 2.2.1. Binding Affinities for the Human SST_2_R

**Figure 2:**
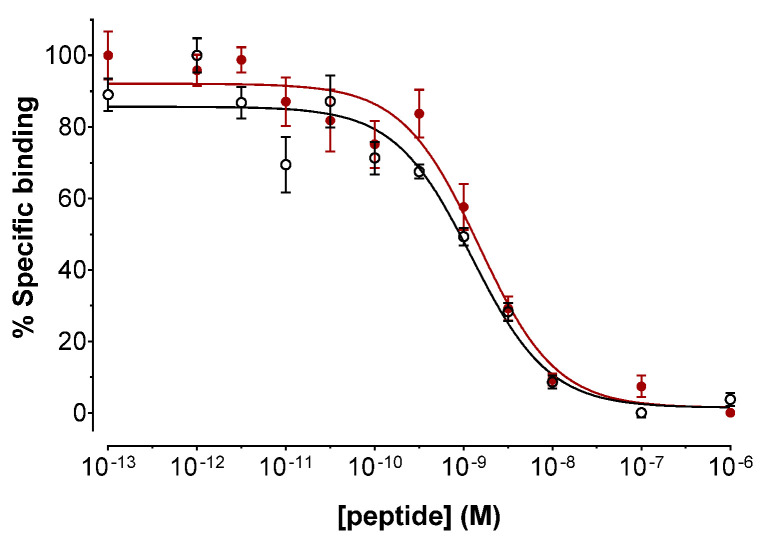
Displacement of [^125^Tyr^25^]LTT-SS28 from SST_2_R binding sites in HEK293-SST_2_R cell membrane homogenates by increasing concentrations of: ○ DATA^5m^-LM4 (IC_50_ = 1.24 ± 0.20 nM, *n* = 3); ● [^nat^Ga]Ga-DATA^5m^-LM4 (IC_50_ = 1.61 ± 0.32 nM, *n* = 3); results represent the average IC_50_ values ± sd, *n* is the number of independent experiments performed in triplicate.

The binding affinities of DATA^5m^-LM4 and [^nat^Ga]Ga-DATA^5m^-LM4 for the human SST_2_R were determined via competition binding assays against [^125^I-Tyr^25^]LTT-SS28 in freshly harvested HEK293-SST_2_R cell membranes [28,30,33]. As depicted in Figure 2, DATA^5m^-LM4 as well as its [^nat^Ga]Ga-tagged version displaced [^125^I-Tyr^25^]LTT-SS28 from SST_2_R-binding sites in the membrane homogenates in a monophasic and dose-dependent manner. Their affinities for the SST_2_R were comparable, with IC_50_ values (mean ± standard deviation (sd), *n* = 3) of 1.24 ± 0.20 nM for DATA^5m^-LM4 and 1.61 ± 0.32 nM for [^nat^Ga]Ga-DATA^5m^-LM4 (*p* > 0.05). 

#### 2.2.2. Uptake/Internalization in HEK293-SST_2_R Cells

**Figure 3:**
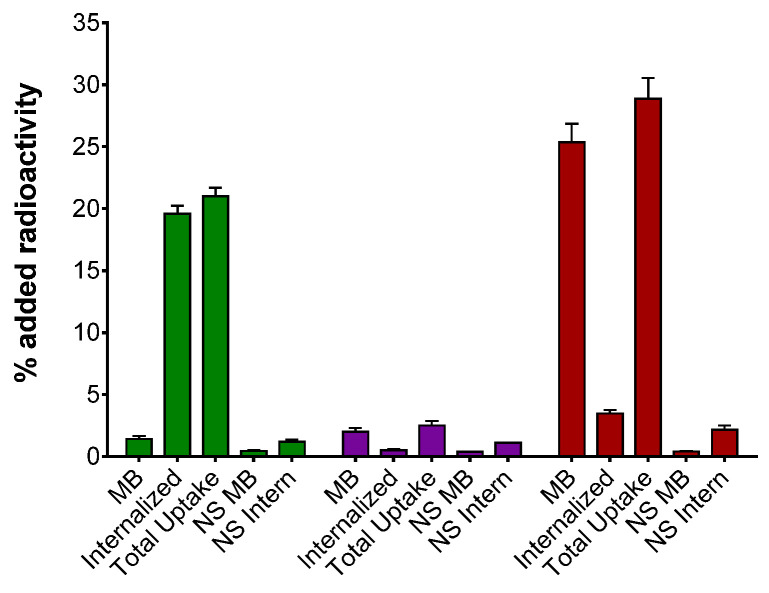
Uptake/internalization of ■ [^67^Ga]Ga-DOTA-TOC, ■ [^67^Ga]Ga-DOTA-LM3 and ■ [^67^Ga]Ga-DATA^5m^-LM4 after 1 h incubation in HEK293-SST_2_R cells at 37 °C; results represent the average values ± sd, acquired from 3 independent experiments performed in triplicate; MB: membrane bound, total uptake: MB + internalized, NS MB: non-specific membrane bound and NS Intern: non-specific internalized; non-specific values were determined in the presence of 1 μM TATE.

Comparative uptake and internalization of [^67^Ga]Ga-DOTA-TOC, [^67^Ga]Ga-DOTA-LM3 and [^67^Ga]Ga-DATA^5m^-LM4 in HEK293-SST_2_R after 1 h at 37 °C are summarized in Figure 3. We observe that [^67^Ga]Ga-DATA^5m^-LM4 is taken up by the cells most efficiently (28.86 ± 1.69%), followed by [^67^Ga]Ga-DOTA-TOC (20.98 ± 0.71%; *p* < 0.0001) and [^67^Ga]Ga-DOTA-LM3, which achieved very low cell uptake (2.51 ± 0.36%; *p* < 0.0001) within this group of radioligands. Notably, the bulk of radioactivity was found in the cell membrane in the case of [^67^Ga]Ga-DATA^5m^-LM4 (25.39 ± 1.48% membrane-bound fragment), whereas for [^67^Ga]Ga-DOTA-TOC, it already internalized within the cells (19.57 ± 0.64% internalized fragment). This distinct cell distribution pattern between [^67^Ga]Ga-DATA^5m^-LM4 and [^67^Ga]Ga-DOTA-TOC is consistent with an SST_2_R antagonist and agonist behavior, respectively [14,16]. In all cases, cell uptake was banned in the presence of excess TATE, revealing an SST_2_R-mediated process.

### 2.3. Animal Studies

#### 2.3.1. In Vivo Metabolic Stability of [^67^Ga]Ga-DATA^5m^-LM4

The in vivo formation of radio metabolites following injection of [^67^Ga]Ga-DATA^5m^-LM4 in healthy mice could be ruled out by radio-HPLC analysis of blood samples collected 5 min post-injection (pi) [34], revealing the high metabolic stability of the octapeptide radiotracer (Supplementary Materials Figure S2). 

#### 2.3.2. Comparison of [^67^Ga]Ga-DATA^5m^-LM4 vs. [^67^Ga]Ga-DOTA-LM3 in Tumor-Bearing Mice 

**Figure 4:**
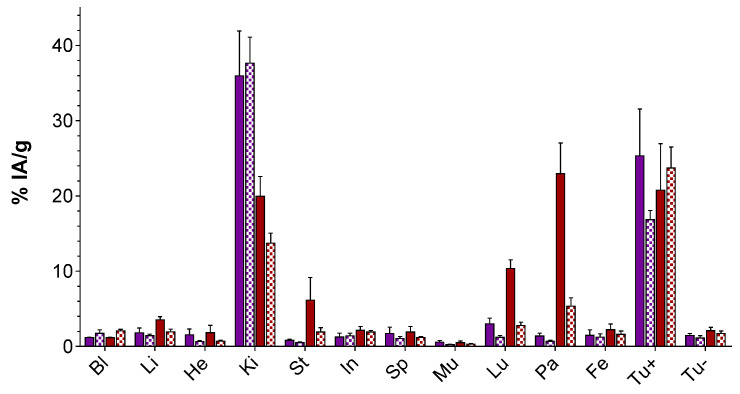
Comparative biodistribution of ■ [^67^Ga]Ga-DOTA-LM3 and ■ [^67^Ga]Ga-DATA^5m^-LM4 in SCID mice bearing twin HEK293-SST_2_R and wtHEK293 xenografts, at 1 h (solid bars) and 4 h (chequered bars) pi. Results are expressed as %IA/g and represent the average values ± sd, *n* = 4; Bl: blood, Li: liver, He: heart, Ki: kidneys, St: stomach, In: intestines, Sp: spleen, Mu: muscle, Lu: lungs, Pa: pancreas, Fe: femur, Tu+: HEK293-SST_2_R tumor, Tu-: wtHEK293 tumor.

The biodistribution patterns of [^67^Ga]Ga-DATA^5m^-LM4 vs. [^67^Ga]Ga-DOTA-LM3 were compared at 1 and 4 h pi in male SCID mice bearing twin HEK293-SST_2_R and wild type (wt)HEK293 tumors in their flanks. Results were calculated as percentage of the injected activity per gram tissue (%IA/g) and represent average values ± sd (*n* = 4); data are shown in Figure 4 (and in numerical values in Table S2, Supplementary Materials).

Both compounds displayed high uptake in the HEK293-SST_2_R tumors at all time intervals and minimal uptake in the wtHEK293 tumors, which were devoid of SST_2_R expression (*p* < 0.0001), in line with a receptor-mediated process. For example, the uptake [^67^Ga]Ga-DATA^5m^-LM4 amounted to 20.76 ± 6.19%IA/g in the HEK293-SST_2_R vs. 2.10 ± 0.43%IA/g in the wtHEK293-SST_2_R xenografts at 1 h pi (*p* < 0.0001). The respective values for [^67^Ga]Ga-DOTA-LM3 were 25.31 ± 6.23%IA/g and 1.45 ± 0.27%IA/g (*p* < 0.0001). Interestingly, the uptake of [^67^Ga]Ga-DATA^5m^-LM4 in the SST_2_R xenografts remained in the same level between 1 and 4 h pi (20.76 ± 6.19%IA/g and 23.70 ± 2.82%IA/g, respectively; *p* > 0.05). In contrast, tumor uptake of [^67^Ga]Ga-DOTA-LM3 unfavorably declined within the same time period (25.31 ± 6.23%IA/g and 16.83 ± 1.22%IA/g, respectively; *p* < 0.0001).

The radioligands displayed fast background clearance predominantly via the kidneys and the urinary tract. Interestingly, [^67^Ga]Ga-DATA^5m^-LM4 showed higher uptake in a number of SST_2_R-positive mice tissues at 1 h pi compared with [^67^Ga]Ga-DOTA-LM3, such as the pancreas (22.96 ± 4.06%IA/g vs. 1.49 ± 0.71%IA/g for [^67^Ga]Ga-DOTA-LM3; *p* < 0.0001) and the stomach (6.16 ± 3.00%IA/g vs. 0.80 ± 0.17%IA/g for [^67^Ga]Ga-DOTA-LM3; *p* < 0.001). These discrepancies however drastically diminished at 4 h pi. A further striking difference in the biodistribution of the two analogs was observed in their kidney uptake and retention. Thus, for [^67^Ga]Ga-DATA^5m^-LM4 the lower renal uptake declined between 1 and 4 h pi (19.94 ± 2.63%IA/g to 13.72 ± 1.33%IA/g, *p* < 0.001) as opposed to [^67^Ga]Ga-DOTA-LM3, which was strongly retained in the kidneys within this time frame (35.95 ± 5.96%IA/g to 37.65 ± 3.44%IA/g, *p* > 0.05), with a more attractive pharmacokinetic profile assigned to the new radiotracer. 

### 2.4. PET/CT of a NET Patient

**Figure 5:**
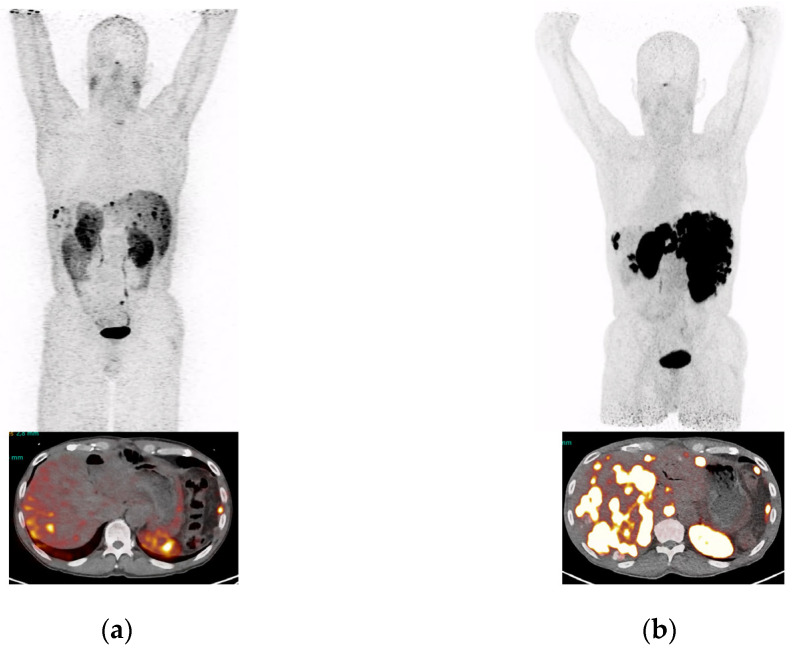
MIPs and transversal images of a patient with pancreatic NET, presenting hepatic and peritoneal lesions 1 h after intravenous injection of the radioligand. (**a**) [^64^Cu]Cu-DOTA-TATE PET/CT, (**b**) [^68^Ga]Ga-DATA^5m^-LM4 PET/CT of the patient having received several treatments including several cycles of PRRT with [^177^Lu]Lu-DOTA-TATE, but remaining with progressive disease.

A 60-year old patient with well-differentiated, functioning NET of the pancreas with extensive hepatic and peritoneal metastases, a Ki-67 index of 52% G3, and was MSI positive, was imaged in November 2021 with [^68^Ga]Ga-DATA^5m^-LM4. He was previously treated with Whipple operation, CAPTEM chemotherapy, segmental liver resection, trans-arterial chemoembolization of metastases in the right liver lobe, Sunitinib, Everolimus, Pembrolizumab, Temodal and Sandostatin. The patient had received a total of 6 cycles of PRRT, cumulative administered radioactivity of 44 GBq Lu-177. At restaging in November 2021 with [^68^Ga]Ga-DATA^5m^-LM4 PET/CT (Figure 5b) and prior to his 5th and 6th cycle, the patient showed progressive disease consistent with massive progression of liver metastases, not seen on [^64^Cu]Cu-DOTA-TATE PET/CT in April 2021 (Figure 5a). 

## 3. Discussion

We herein introduce [^68^Ga]Ga-DATA^5m^-LM4, as an SST_2_R antagonist in the diagnosis of NETs with PET/CT. The new tracer is based on the known SST_2_R antagonist LM3 [21,22] following 4Pal^3^/Tyr^3^-substitution [32] in the cyclic octapeptide chain. Notably, DATA^5m^-LM4 can be labeled with Ga-67/68 at much lower temperatures than other DOTA-derivatized peptides (including DOTA-LM3) by virtue of the hybrid DATA^5m^ chelator attached on its N-terminus (Figure 1) [25,26,27,28,29,30]. For convenient completion of the preclinical study, DATA^5m^-LM4, DOTA-LM3 and the SST_2_R-agonist reference DOTA-TOC were labeled with the longer-lived Ga-67 (t_1/2_: 78.3 h) as a Ga-68 surrogate (t_1/2_: 67.7 min). Unlike the above two DOTA-conjugates requiring heating at 90 °C for 30 min during labeling, DATA^5m^-LM4 was successfully labeled by a mere 15 min incubation at room temperature. The quality control of radiolabeled products adopting radioanalytical HPLC methods confirmed in all cases a >98% RCP at a molar activity of 3.7 MBq/nmol. Therefore, all radioligands were further used without purification in all preclinical experiments that followed.

During competition binding assays against [^125^I-Tyr^25^]LTT-SS28 on HEK293-SST_2_R cell-membranes [30], DATA^5m^-LM4 and its Ga-tagged version [^nat^Ga]Ga-DATA^5m^-LM4 showed high receptor affinity, reflected in the single-digit nanomolar IC_50_ values (1.24 ± 0.20 nM and 1.61 ± 0.32 nM, respectively; Figure 2). It is interesting to note that the IC_50_ values reported for DOTA-LM3 and [^nat^Ga]Ga-DOTA-LM3 during SST_2_R autoradiography against the same radioligand were 1.4 ± 0.5 nM and 12.5 ± 4.3 nM, respectively [15,22]. On the other hand, by changing the metal to Lu the IC_50_ determined for [^nat^Lu]Lu-DOTA-LM3 was 1.61 ± 0.32 nM and by replacing DOTA by NODAGA, the IC_50_ value for [^nat^Ga]Ga-NODAGA-LM3 reached 1.3 ± 0.3 nM [21,22]. These results reveal the strong impact of the metal chelate on SST_2_R affinity. Thus, Ga-labeling of DOTA-LM3 leads to a loss of receptor binding affinity compared with unlabeled DOTA-LM3 by one order of magnitude. In turn, these data reveal that the binding affinity of Ga-labeled DATA^5m^-LM4 is 10-fold better than that of Ga-labeled DOTA-LM3, while both unlabeled compounds show similar binding affinity. Specific uptake of the [^67^Ga]Ga-radioligands in HEK293-SST_2_R cells followed this affinity pattern (Figure 3), with [^67^Ga]Ga-DATA^5m^-LM4 taken up much more successfully by the cells at 1 h incubation compared with [^67^Ga]Ga-DOTA-LM3 (28.86 ± 1.69 % vs. 2.51 ± 0.36%, respectively). It is also interesting to compare the cell distribution pattern of radioactivity between [^67^Ga]Ga-DATA^5m^-LM4 with the radioactivity predominantly found on the cell membrane and [^67^Ga]Ga-DOTA-TOC with the bulk of radioactivity internalized. These two distinct patterns correspond to a non-internalizing receptor antagonist and an internalizing agonist profile.

The biodistribution of [^67^Ga]Ga-DATA^5m^-LM4 and [^67^Ga]Ga-DOTA-LM3 were directly compared in male SCID mice bearing twin HEK293-SST_2_R and wtHEK293 xenografts in their flanks, with the latter serving as negative controls being devoid of SST_2_R expression. The new tracer [^67^Ga]Ga-DATA^5m^-LM4 showed a favorably constant uptake in the implanted HEK293-SST_2_R tumors in the period between 1 and 4 h pi, as opposed to [^67^Ga]Ga-DOTA-LM3 declining from the tumor within this interval. Another interesting advantage of [^67^Ga]Ga-DATA^5m^-LM4 is the significantly lower kidney uptake at 1 h pi, which further decreased at 4 h pi. In contrast, [^67^Ga]Ga-DOTA-LM3 retained a much higher kidney uptake within this time frame (Figure 4; Table S2, Supplementary Materials). It is interesting to note that biodistribution results on [^67^Ga]Ga-DOTA-LM3 at 1 h pi are well comparable with those previously reported for [^68^Ga]Ga-DOTA-LM3 on a similar HEK293-SST_2_R tumor model in female nude mice; the same total peptide amount (10 pmol) was injected in both cases [22]. Summarizing the above observations, [^67^Ga]Ga-DATA^5m^-LM4 displayed i. lower renal uptake than [^67^Ga]Ga-DOTA-LM3 at all time points, ii. stable tumor uptake vs. the decreasing tumor uptake of [^67^Ga]Ga-DOTA-LM3, resulted in an overall better profile. Thus, tumor-to-kidney ratios increased over time for [^67^Ga]Ga-DATA^5m^-LM4 (1.04 at 1 h up to 1.73 at 4 h pi), as opposed to [^67^Ga]Ga-DOTA-LM3 showing decreasing tumor-to-kidney ratios within this time frame (0.70 at 1 h down to 0.45 at 4 h pi).

The above discussed promising preclinical results prompted us to test clinical grade [^68^Ga]Ga-DATA^5m^-LM4 in a NET patient in a preliminary “proof-of-principal” experiment. It should be noted that routine labeling with Ga-68 was successful and reproducible via an automated module in the hospital and mild heating at 50 °C in an overall production time of 15 min. Notably, tumor lesions were accurately visualized in the patient with a high contrast on PET/CT (Figure 5).

The present study has revealed attractive features for [^67^Ga]Ga-DATA^5m^-LM4 firstly at the preclinical level, such as high receptor affinity and cell uptake, in vivo robustness as well as high and sustained uptake in SST_2_R-positive tumors in mice combined with a favorable washout from the kidneys into the urine. These excellent qualities were replicated in a NET patient injected with clinical grade of [^68^Ga]Ga-DATA^5m^-LM4 applying PET/CT. Labeling of the new agent was fast, simple and reproducible for both preclinical and clinical preparations via the DATA^5m^-chelator route. Further systematic clinical studies are warranted to establish the diagnostic value of [^68^Ga]Ga-DATA^5m^-LM4 in the detection of NETs with PET/CT.

## 4. Materials and Methods

### 4.1. Chemicals, Ligands and Radionuclides

Common chemicals used were reagent grade except for HPLC solvents which were HPLC grade. DOTA-TOC (TOC, [Tyr^3^]octreotide: DPhe-c[Cys-Tyr-DTrp-Lys-Thr-Cys]-Thr-ol; DOTA: 1,4,7,10-tetraazacyclododecane-1,4,7,10-tetraacetic acid) was purchased from ABX Advanced Biochemical Compounds, GmbH (Radeberg, Germany). DOTA-LM3 (LM3: p-Cl-Phe-c[DCys-Tyr-D-4-(carbamoyl)amino-Phe-Lys-Thr-Cys]-DTyr-NH_2_) was provided by PiChem Forschungs- und Entwickungs GmbH (Raaba-Grambach, Austria) and DATA^5m^-LM4 (LM4: p-Cl-Phe-c[DCys-(4-pyridyl)Ala-D-4-(carbamoyl)amino-Phe-Lys-Thr-Cys]-DTyr-NH_2_); DATA^5m^: (6-pentanoic acid)-6-(amino)methy-1,4-diazepinetriacetate) was obtained from Peptide Specialty Laboratories GmbH (Heidelberg Germany); analytical data of the two conjugates, comprising MALDI-TOF mass spectroscopy results and purity determined by HPLC analysis is compiled in Table S1 (Supplementary File). TATE (H-DPhe-c[Cys-Tyr-DTrp-Lys-Thr-Cys]-Thr-OH) was synthesized on the solid support, as previously described [28]. LTT-SS28 (H-Ser-Ala-Asn-Ser-Asn-Pro-Ala-Leu-Ala-Pro-Arg-Glu-Arg-Lys-Ala-Gly-c[Cys-Lys-Asn-Phe-Phe-DTrp-Lys-Thr-Tyr-Thr-Ser-Cys]-OH) was obtained from Bachem AG (Bubbendorf, Switzerland). [^nat^Ga]Ga(NO_3_)_3_ was purchased from Sigma-Aldrich Inc. (St. Louis, MO, USA).

Gallium-67 in the form of a [^67^Ga]GaCl_3_ solution in dilute HCl was provided by IDB Holland BV (Baarle-Nassau, The Netherlands) and used as a surrogate of Ga-68 in preclinical studies. For the clinical preparation, Ga-68 from a pharmaceutical grade commercial [^68^Ge]Ge/[^68^Ga]Ga-generator (GalliaPharm^®^ from Eckert & Ziegler Strahlen- und Medizintechnik AG, Berlin, Germany) was employed. For the preparation of [^125^I-Tyr^25^]LTT-SS28, [^125^I]NaI was provided by PerkinElmer (Waltham, MA, USA) in dilute sodium hydroxide solution (pH 8–11). 

#### 4.1.1. Radiolabeling

Lyophilized peptide conjugates were dissolved in HPLC-grade H_2_O and distributed in 50 μL aliquots in Eppendorf Protein LoBind tubes. These were stored at −20 °C. For labeling with Ga-67, each conjugate (5 nmol) was mixed with 17–20 MBq of [^67^Ga]GaCl_3_ and 1 M sodium acetate was added to adjust the pH of the reaction to 4.0. The mixture was incubated at room temperature for 15 min for [^67^Ga]Ga-DATA^5m^-LM4 and at 90 °C for 30 min for [^67^Ga]Ga-DOTA-TOC and [^67^Ga]Ga-DOTA-LM3. Sodium EDTA (0.1 M, pH 4.0) was added to a final concentration of 1 mM as a scavenger of “free” [^67^Ga]Ga^3+^ traces [28].

Clinical grade [^68^Ga]Ga-DATA^5m^-LM4 was obtained via an automatic mini all-in-one cassette-based module from Trasis (Ans, Belgium). Labeling with Ga-68 proceeded at 50 °C in sodium acetate buffer (0.7 M, pH 5.5, 1 mL) resulting in a >98 RCP within 10 min reaction time [27,29]. The automated synthesis yielded activities between 413 MBq and 596 MBq (avg. 508 ± 86 MBq; *n* = 9) after 15 min of production with 50 μg DATA^5m^-LM4 and 20 min of quality control.

The [^125^I][I-Tyr^25^]LTT-SS28 was obtained from LTT-SS28 according to the chloramine-T method using [^125^I]NaI in a 0.1 M NaOH solution for the radioiodination [30]. The radioligand was isolated in a pure form by HPLC and aliquots thereof in 0.1% BSA-PBS buffer were kept at −20 °C; these were used for competition binding experiments (molar activity of 74 GBq/μmol). For the preparation of [^nat^Ga]Ga-DATA^5m^-LM4, stock solution of DATA^5m^-LM4 (60 µL, 2 mM, 120 nmol) was placed in an Eppendorf Protein LoBind^®^ centrifuge tube and a [^nat^Ga]Ga(NO_3_)_3_ solution in 1 M sodium acetate buffer (pH 4.0) was added. The mixture was heated at 75 °C for 1 h and analysis was conducted applying reversed-phase high performance liquid chromatography (RP-HPLC).

#### 4.1.2. Radiochemical Analysis

Analyses were performed on a Waters Chromatograph based on a 600E multi-solvent delivery system applying twin detection modes: (a) for photometric detection, a Waters 2998 photodiode array detector (Waters, Vienna, Austria) was used (DATA^5m^-LM4 and [^nat^Ga]Ga-DATA^5m^-LM4), (b) for radiometric detection, a Gabi gamma-detector (Raytest, RSM Analytische Instrumente GmbH, Straubenhardt, Germany) was applied (detection of radioligands). The system was monitored and data was processed by the Empower Software (Waters, Milford, MA, USA). For radiochemical analyses, samples were eluted through a Xterra RP18 cartridge column (5 μm, 3.9 mm × 20 mm, Waters, Eschborn, Germany), applying the following linear gradient: 100%A/0% B to 60%A/40% B in 40 min, whereby A = 0.1% TFA in H_2_O (*v*/*v*) and B = MeCN (system 1). Radioligands were used without further purification in all subsequent experiments and suitable samples were tested before and after the end of all biological experiments. Retention times (*t*_R_) were as follows: [^67^Ga]Ga-DATA^5m^-LM4: 22.5 min, [^67^Ga]Ga-DOTA-TOC: 21.4 min and [^67^Ga]Ga-DOTA-LM3: 24.7 min.

The handling of solutions containing beta-/gamma-emitting radionuclides was conducted by authorized personnel in compliance with European radiation safety guidelines. Licensed facilities were supervised by the Greek Atomic Energy Commission (GAEC, license #A/435/17092/2019 and #A/435/15767/2019).

For quality control of clinical grade [^68^Ga]Ga-DATA^5m^-LM4, radio-TLC was applied (TLC silica gel 60 F254 Merck) whereby the plates were developed with (1) 0.1 M citrate buffer pH 4 and (2) a 1:1 mixture of 1 M ammonium acetate and MeOH (v:v) as the mobile phase. TLC plates were measured on a miniGita TLC scanner from Elysia-Raytest (Angleur, Belgium) applying the GINA analysis software (Elysia-Raytest; Angleur, Belgium). Radio-HPLC was conducted on an Infinity 1200 analytical HPLC system from Agilent Technologies (Waldbronn, Germany), coupled to a Ramona * radiodetector from Elysia-Raytest (Angleur, Belgium). The VDSpher PUR 150 C18-E column (5 μm, 100 × 40 m) was eluted applying a linear gradient of 5–45% MeCN (+0.1% TFA)/95–55% H_2_O (+0.1% TFA) in 10 min (system 2). No indices for tracer instability were detected in the period between synthesis and image acquisition.

### 4.2. Cell Studies

#### 4.2.1. Cell Culture

The HEK293 cell line transfected to stably express the human SST_2_R, tagged with the T7-epitope (HEK293-SST_2_R), was offered by S. Schultz (Jena, Germany) [33]. Wild type HEK293 cells, devoid of SST_2_R expression, served as negative controls (wtHEK293). Cells were cultured in controlled humidified air with 5% CO_2_ at 37 °C in Dulbecco’s Modified Eagle Medium (DMEM), containing Glutamax-I and supplemented with 10% (*v*/*v*) heat-inactivated fetal bovine serum (FBS), 100 U/mL penicillin, 100 μg/mL streptomycin and 400 μg/mL G418; the latter was not added during culturing of wtHEK293 cells. All culture media were provided by Gibco BRL, Life Technologies (Grand Island, NY, USA) and supplements were supplied by Biochrom KG Seromed (Berlin, Germany). Sub-culturing was achieved by treating the cells with a trypsin/EDTA (0.05%/0.02% *w*/*v*) solution.

#### 4.2.2. Competition Binding Assays in HEK293-SST_2_R Cell Membranes

Competition binding assays for DATA^5m^-LM4 and [^nat^Ga]Ga-DATA^5m^-LM4 were performed in freshly harvested HEK293-SST_2_R cell membranes against [^125^I-Tyr^25^]LTT-SS28 [30,33]. In short, to each triplicate of assay tube corresponding to a different concentration point, the following were added: i. the test compound or the reference (30 μL solution of increasing concentrations, 10^−5^–10^−13^ M), ii. the radioligand (70 μL, 50 pM corresponding to ≈ 40,000 cpm) and iii. the membrane homogenate (200 μL) to a final volume of 300 μL in binding buffer (50 mM HEPES pH 7.4, 1% BSA, 5.5 mM MgCl_2_, 35 μM bacitracin). Samples were incubated for 1 h at 22 °C in an Incubator Orbital Shaker unit, (MPM Instr. SrI) and a chilled washing buffer (10 mM HEPES pH 7.4, 150 mM NaCl) was added to stop the reaction. Samples underwent rapid filtration through glass fiber filters (Whatman GF/B, pre-soaked for 2 h in a 1 % polyethyleneimine (PEI) aqueous solution) on a Brandel Cell Harvester (Adi Hassel Ingenieur Büro, Munich, Germany) and were washed with ice-cold washing buffer (10 mM HEPES pH 7.4, 150 mM NaCl). Filters were collected and their activity measured in a γ-counter (automated multi-sample well-type instrument with a NaI(Tl) 3″ crystal, Canberra Packard Cobra^TM^ Quantum U5003/1, Auto-Gamma^®^ counting system). The half-maximal inhibitory concentration (IC_50_) values were calculated by nonlinear regression for a one-site model applying the PRISM^TM^ 6.0 GraphPad software (San Diego, CA, USA) and represent mean IC_50_ ± sd values from at least three independent experiments were performed in triplicate.

#### 4.2.3. Radioligand Uptake/Internalization in HEK293-SST_2_R Cells

The uptake/internalization of [^67^Ga]Ga-DOTA-TOC, [^67^Ga]Ga-DOTA-LM3 and [^67^Ga]Ga-DATA^5m^-LM4 were compared by 1 h incubation in HEK293-SST_2_R cells at 37 °C. Cells were seeded in poly-lysine coated six-well plates (1 × 10^6^/well) and grew to confluent monolayers overnight. At the day of the experiment, plates were placed on ice and rinsed twice with chilled internalization medium (DMEM Glutamax-I supplemented by 1% (*v*/*v*) FBS). Fresh medium was added (1.2 mL) at room temperature, followed by a solution of [^67^Ga]Ga-DOTA-TOC, [^67^Ga]Ga-DOTA-LM3 or [^67^Ga]Ga-DATA^5m^-LM4 (50,000 cpm corresponding to 0.5 pmol total peptide in 150 μL 0.5% BSA PBS). In the upper three wells internalization medium was added (150 μL, total) and in the lower three a solution of TATE to a final concentration of 1 μM (150 μL, non-specific). After 60 min incubation at 37 °C, the medium was removed and cells were washed with ice-cold 0.5% BSA-PBS. Cells were incubated (2 × 5 min) at ambient temperature in acid wash buffer (50 mM glycine in 0.1 M NaCl, pH 2.8) and supernatants were collected (membrane-bound fraction). After rinsing with 0.5% BSA-PBS, the cells were lysed by treatment with 1 N NaOH; the lysates were collected (internalized fraction). Samples were measured in the γ-counter and cell-associated/internalized radioactivity vs. total added activity per well was calculated. Specific values were obtained by subtracting values in the presence of excess TATE (lower well triplicates) from those without the addition of blocker (upper well triplicates). Results represent average ± sd values from at least three independent experiments.

### 4.3. Animal Studies

#### 4.3.1. Stability Studies

The metabolic stability of [^67^Ga]Ga-DATA^5m^-LM4 was assessed in three healthy male Swiss albino mice provided by the NCSR “Demokritos” Animal House (Athens, Greece; body weight: 30 ± 5 g). Mice were injected through their tail vein with a 100-μL bolus containing the radioligand (up to 11 MBq corresponding to 2.5–3 nmol of total conjugate in vehicle: saline/EtOH 9/1 *v*/*v*) and were euthanized 5 min post-injection (pi). Blood was directly withdrawn from the heart in a pre-chilled syringe and placed in an ice-cold Eppendorf Protein LoBind^®^ tube on ice containing EDTA (40 µL, 50 mM Na_2_EDTA solution). Samples were centrifuged (10 min, 2000× *g*/4 °C, in a Hettich Universal 320R centrifuge, Tuttlingen, Germany), the plasma was collected and cold MeCN was added in a 1/1 *v*/*v* ratio. Samples were centrifuged once more (10 min, 15,000× *g*/4 °C) and supernatants were concentrated to a small volume (≈50–100 μL) under a gentle N_2_-flux at 40 °C. Physiological saline (400 μL) was added and the solution was passed through a Millex GV filter (0.22 μm, 13 mm Ø, Millipore, Milford, MI, USA). Filtrate samples were analyzed by HPLC (system 1) [34]. The elution time (*t*_R_) of intact [^67^Ga]Ga-DATA^5m^-LM4 was determined by co-injection of blood samples with an aliquot of the labeling solution. Results as average percentage of intact radioligand ± sd were acquired from three mice.

#### 4.3.2. Biodistribution in SCID Mice Bearing Twin HEK293-SST_2_R and wtHEK293 Tumors

Inocula containing a suspension of freshly-harvested HEK293-SST_2_R cells (150 μL, 1.2 × 10^7^cells in normal saline) and wtHEK293 cells (150 μL, 0.6 × 10^7^ cells in normal saline) were subcutaneously (sc) injected in the right and left flanks of male SCID mice (21.5–25.0 g body weight, six weeks of age on arrival day; NCSR “Demokritos” Animal House, Athens, Greece). Mice were kept under aseptic conditions for about 2 weeks until well-palpable tumors (300–600 mg) were grown at the inoculation sites [28]. On the day of biodistribution, animals received a 100 μL-bolus through the tail vein containing [^67^Ga]Ga-DOTA-LM3 or [^67^Ga]Ga-DATA^5m^-LM4 (37 kBq, 10 pmol total peptide) and were euthanized in groups of four at 1 and 4 h pi. Samples of blood and tissues of interest were collected along with implanted tumors, weighted and counted in the gamma counter. Biodistribution results were calculated as percent of the injected activity per gram tissue (%IA/g) with the aid of appropriate standards of the administered dose and the Microsoft Excel program. Results are presented as mean %IA/g values ± sd, *n* = 4 per time point, as determined by the PRISM^TM^ 6.0 GraphPad software (San Diego, CA, USA).

Mice experiments complied with European and national regulations in licensed facilities (EL 25 BIO exp021). The study protocols were approved by the Department of Agriculture and Veterinary Service of the Prefecture of Athens (#1609, 24-04-2019 for the stability studies and #1610, 24-04-2019 for the biodistribution and imaging studies).

#### 4.3.3. Statistical Analysis

For statistical comparison of the results, a two-way ANOVA with multiple comparisons and Tukey’s post-hoc analysis was applied (PRISM^TM^ 6.0 GraphPad Software, San Diego, CA, USA). *p* values of < 0.05 were considered to be statistically significant.

### 4.4. Patient Study

The patient signed written informed consent before the study. He was intravenously injected with a dose of [^68^Ga]Ga-DATA^5m^-LM4 adopted to his body weight (1.8–2.2 MBq/kg). PET/CT images were acquired 1 h pi from the vertex to the proximal femora on a Biograph Vision 600 Edge PET/CT scanner (Siemens Healthineers AG; Erlangen, Germany). A low-dose CT scan from head to the proximate thigh was obtained for attenuation correction and anatomical mapping. Next, whole-body PET was performed with 2 min per bed position (5–7 bed positions depending on the height of the patient). The patient had a good renal function and showed no allergies to any of the ingredients of the radiopharmaceuticals. PET/CT images were anonymized and reviewed by two experienced nuclear medicine experts who were not blinded to patient medical history on the reconstructed images.

## Data Availability

Not applicable.

## Supplementary Materials

The following supporting information can be downloaded at: https://www.mdpi.com/article/10.3390/ijms232314590/s1.
