# Supplementary material for: [68Ga]Ga-DATA5m-LM4, a PET Radiotracer in the Diagnosis of SST2R-Positive Tumors: Preclinical and First Clinical Results
Source: Int J Mol Sci. 2022 Nov 23;23(23):14590. doi: 10.3390/ijms232314590 (PMC9740503; doi:10.3390/ijms232314590)
Supplement: Supplementary file 1 [file ijms-23-14590-s001.zip › ijms-2047761-supplementary.pdf]

International Journal of *Molecular Sciences*

Article

Supplementary Materials: [68Ga]Ga-DATA5m-LM4, a PET Radiotracer in the Diagnosis of SST2R-Positive Tumors: Preclinical and First Clinical Results

Panagiotis Kanellopoulos, Berthold A. Nock, Lukas Greifenstein, Richard P. Baum, Frank Roesch and Theodosia Maina*

Analytical data for DATA5m-LM4 and DOTA-LM3

Analytical data for DATA5m-LM4 (Peptide Specialty Laboratories GmbH; Heidelberg Germany) and DOTA-LM3 (PiChem Forschungs- und Entwickungs GmbH; Raaba-Grambach, Austria), comprising purity via HPLC analysis and MALDI-TOF results are compiled in Table S1.

Table S1. Analytical data for DATA5m-LM4 and DOTA-LM3 in SCID.

Compound	Structure	% Purity	MW[c] calcd MW[c] found, m/z
DATA5m-LM4	DATA5m-p-Cl-Phe-c[DCys-4Pal-DAph(Cbm)-Lys-Thr-Cys]-DTyr-NH2 [a]	≥ 95	1532.60<br>1531.32
DOTA-LM3	DOTA-p-Cl-Phe-c[DCys-Tyr-DAph(Cbm)-Lys-Thr-Cys]-DTyr-NH2 [b]	≥ 95	1548.60<br>1550.16

[a] DATA5m: (6-pentanoic acid)-6-(amino)methy-1,4-diazepinetriacetate, 4-Pal; (4-pyridyl)alanine, DAph(Cbm): D-4-(carbamoyl)amino-phenylalanine; [b] DOTA: 1,4,7,10-tetraazacyclododecane-N,N′,N'',N'''-tetraacetic acid; [c] exact mass.

Representative HPLC Analysis of [67Ga]Ga-DATA5m-LM4 Preparation

A typical radiochromatogram of HPLC analysis applied in the quality control of [67Ga]Ga-DATA5m-LM4 labeled product for preclinical testing is presented in Figure S1. Labeling protocols and HPLC details (system 1) have been reported in the manuscript.

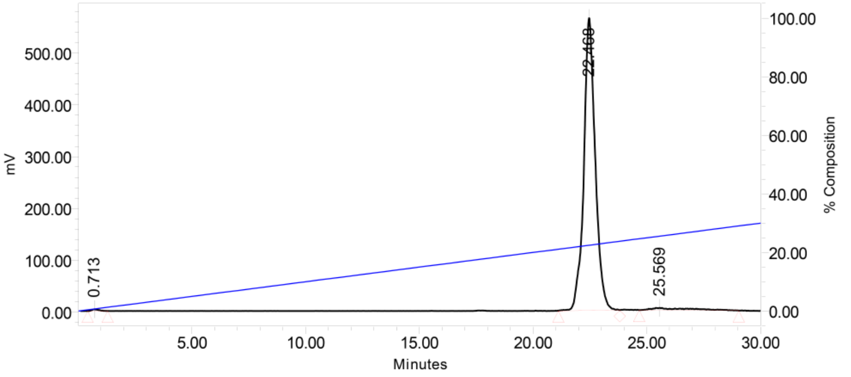

Figure S1. Representative radiochromatogram of [67Ga]Ga-DATA5m-LM4 labeled product confirming the high RCP (99%) formation of the radioligand (retention time (t_R) = 22.4 min on system 1).

International Journal of
Molecular Sciences

Stability of [^{67}Ga]Ga-DATA^{5m}-LM4 in Mice

As detailed in the main manuscript, [^{67}Ga]Ga-DATA^{5m}-LM4 was injected in 3 healthy mice and 5 min pi blood was collected and properly processed for HPLC analysis (system 1) for the detection of radiometabolites. A representative radiochromatogram of HPLC analysis of such sample is shown in Figure S2.

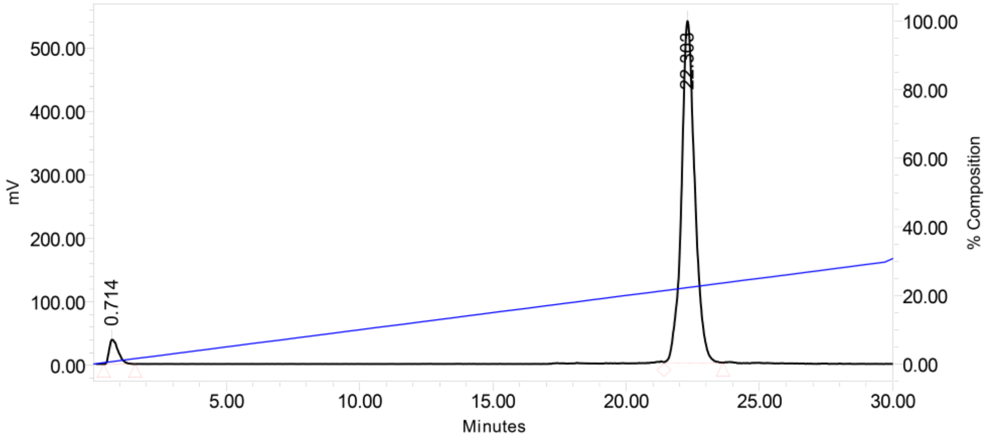

Figure S2. Gamma trace of HPLC analysis of blood samples collected 5 min pi of [^{67}Ga]Ga-DATA^{5m}-LM4 in healthy mice, showing the high stability of the radiotracer in mice circulation (>95% intact); the t_R = 22.3 min on system 1 was determined by co-injection with a labeling reaction sample on the HPLC.

Biodistribution of [^{67}Ga]Ga-DATA^{5m}-LM4 and [^{67}Ga]Ga-DOTA-LM3 in Tumor-Bearing Mice

Results from the biodistribution comparison of [^{67}Ga]Ga-DATA^{5m}-LM4 and [^{67}Ga]Ga-DOTA-LM3 in male SCID mice bearing twin HEK293-SST2R and "mock" wtHEK293 xenografts are summarized in Table S1, as %IA/g numerical values for the 1 h and 4 h pi time intervals, representing average values± sd, n = 4. Experimental details and critical comments on biodistribution outcomes are provided in the main manuscript.

Table S2. Comparative biodistribution data of [^{67}Ga]Ga-DATA^{5m}-LM4 and [^{67}Ga]Ga-DOTA-LM3 in SCID mice bearing twin HEK293-SST$_2$R (Tu+) and wtHEK293 (Tu-) xenografts at 1 h and 4 h pi; data is expressed as %IA/g and represent average values ± sd, n = 4.

Organs/Tissues	[^{67}Ga]Ga-DATA^{5m}-LM4		[^{67}Ga]Ga-DOTA-LM3	
	1 h	4 h	1 h	4 h
Blood	1.17±0.04	2.08±0.20	1.18±0.03	1.73±0.47
Liver	3.55±0.41	1.94±0.37	1.82±0.63	1.43±0.19
Heart	1.82±0.99	0.71±0.14	1.54±0.78	0.66±0.14
Kidneys	19.94±2.63****	13.72±1.33****	35.95±5.96	37.65±3.44
Stomach	6.16±3.00***	1.93±0.55	0.80±0.17	0.50±0.08
Intestines	2.15 ±0.49	1.92±0.19	1.26±0.52	1.41±0.37
Spleen	1.93±0.73	1.88±0.12	1.73±0.85	1.06±0.25
Muscle	0.49±0.22	0.30±0.07	0.56±0.22	0.25±0.06
Lungs	10.36±1.17	2.79±0.44	3.00±0.77	1.21±0.24
Femur	2.24±0.75	1.60±0.47	1.39±0.39	0.67±0.14
Pancreas	22.96±4.06****	5.36±1.08	1.49±0.71	1.24±0.44
Tu+	20.76±6.19*	23.70±2.82***	25.31±6.23	16.83±1.22
Tu-	2.10±0.43	1.73±0.32	1.45±0.27	1.13±0.31

Statistical significant differences between the two radioligands at the same time intervals, ****, $P<0.0001$, ***, $P<0.001$ and *, $P<0.05$.

Quality Control of Clinical Grade [^{68}Ga]Ga-DATA^{5m}-LM4 for Patients

A diagram of the automated set-up used in the hospital for the preparation of clinical grade [^{68}Ga]Ga-DATA^{5m}-LM4 for injection to patients, along with representative examples of quality control TLC and HPLC (system 2) radioanalytical tests are presented in Figure S3.

International Journal of
Molecular Sciences

MDPI

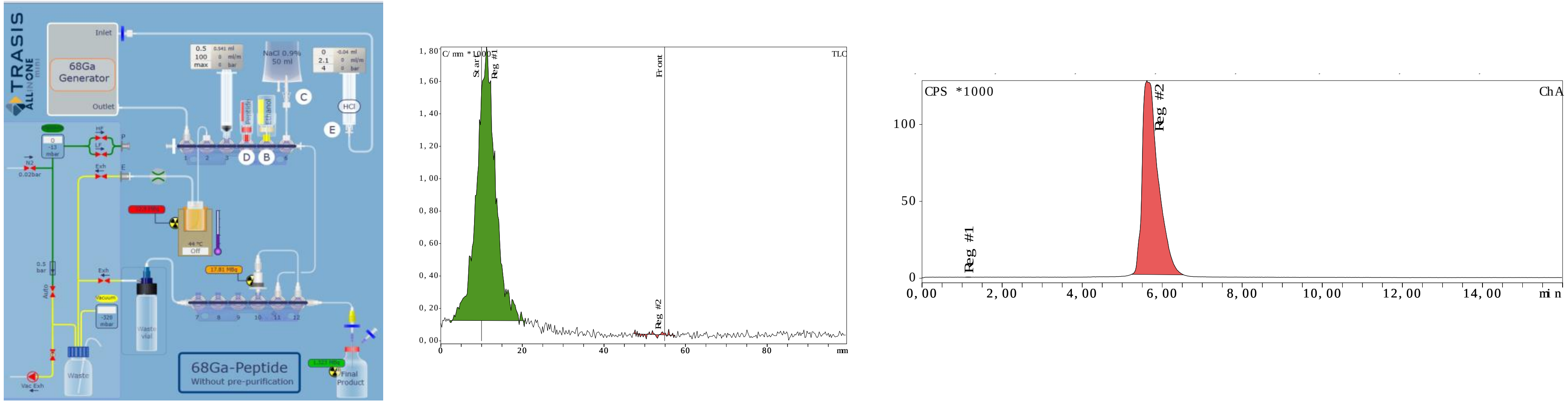

(a) (b) (c)

Figure S3. (**a**) Set-up of the automated production of clinical grade [^{68}Ga]Ga-DATA^{5m}-LM4; (**b**) Typical radioanalytical TLC developed in citric acid buffer; (**c**) Representative radiochromatogram of HPLC analysis of clinical grade [^{68}Ga]Ga-DATA^{5m}-LM4 product (system 2).
